# Uteroglobin and FLRG concentrations in aqueous humor are associated with age in primary open angle glaucoma patients

**DOI:** 10.1186/s12886-018-0723-4

**Published:** 2018-02-27

**Authors:** Esther L. Ashworth Briggs, Tze’Yo Toh, Rajaraman Eri, Alex W. Hewitt, Anthony L. Cook

**Affiliations:** 1 School of Health Sciences, University of Tasmania, Launceston, Australia; 2 Launceston Eye Institute and Launceston Eye Doctors, Launceston, Australia; 3 Centre for Eye Research Australia, University of Melbourne, Melbourne, Australia; 4 Wicking Dementia Research and Education Centre, University of Tasmania, Hobart, 7001 Australia

**Keywords:** Primary open angle glaucoma, Trabecular meshwork, Aqueous humor, Uteroglobin/SCGB1A1, FLRG

## Abstract

**Background:**

The pathophysiological changes occurring in the trabecular meshwork in primary open angle glaucoma are poorly understood, but are thought to include increased extracellular matrix deposition, trabecular meshwork cell apoptosis, inflammation, trabecular meshwork calcification and altered protein composition of the aqueous humor. Although many proteins are present in aqueous humor, relatively few have been studied extensively, and their potential roles in primary open angle glaucoma are unknown.

**Methods:**

Analyte concentrations in aqueous humor from 19 primary open angle glaucoma and 18 cataract patients were measured using a multiplex immunoassay. Fisher’s exact test was used to assess statistical significance between groups, and correlations of analyte concentrations with age, intraocular pressure, pattern standard deviation, mean deviation, cup-to-disc ratio and disease duration since commencing treatment were tested by Spearman’s method.

**Results:**

CHI3L1, FLRG, HGF, MIF, P-selectin and Uteroglobin were detected in more than 50% of samples of one or both patient groups, some of which have not previously been quantified in aqueous humor. In the glaucoma but not the cataract group, significant correlations were determined with age for Uteroglobin/SCGB1A1 (r_s_ = 0.805, *p* < 0.0001) and FLRG (r_s_ = 0.706, *p* = 0.0007). Furthermore, HGF correlated significantly with disease duration (r_s_ = − 0.723, *p* = 0.0007). There were no differences in analyte concentrations between groups, and no other significant associations with clinical descriptors that passed correction for multiple testing.

**Conclusions:**

The correlations of uteroglobin and FLRG with age in primary open angle glaucoma but not cataract may suggest a heightened requirement for anti-inflammatory (uteroglobin) or anti-calcification (FLRG) activity in the ageing glaucomatous trabecular meshwork.

**Electronic supplementary material:**

The online version of this article (10.1186/s12886-018-0723-4) contains supplementary material, which is available to authorized users.

## Background

Aqueous humor is a clear fluid that circulates throughout the anterior chamber of the eye to provide nutrients to and remove metabolic waste products from the tissues it contacts, and thus contributes to the maintenance of normal eye function [1]. The majority of aqueous humor drains from the eye via the trabecular meshwork (TM), a specialised porous tissue responsible for the regulation of intraocular pressure (IOP) [2]. In primary open angle glaucoma (POAG), decreased drainage of aqueous humor through a compromised TM leads to elevated IOP [1], causing optic nerve degeneration and thus a progressive loss of peripheral vision unless treated. Elevated IOP is the only modifiable risk factor for the development of glaucoma, and all current treatments for POAG are aimed at reducing IOP [2].

The molecular and cellular changes that contribute to TM dysfunction and elevated IOP in POAG are poorly understood. Several processes, including altered extracellular matrix (ECM) turnover [3], oxidative stress [4], inflammation [5], reduced TM cellularity [6], increased TM stiffness [7] and TM calcification [8] are all potential contributors to the pathological changes occurring in the TM during POAG. Many clinical studies of glaucomatous aqueous humor samples have reported alterations of multiple inflammatory mediators, including TGF- β2 [9–11], IL-8 [12], IL12, IFNγ and CXCL9 [13, 14] compared to controls, and a pro-inflammatory environment of the aqueous humor has been reported for an animal model of glaucoma [15]. Furthermore, inflammation can cause TM cell apoptosis and lead to a dysfunctional trabecular meshwork, thus contributing to an elevated IOP [5].

Whilst many proteins have been detected in aqueous humor using discovery-based proteomics approaches [16–22], no detailed studies have been performed with regards to these proteins, and thus any potential role in eye physiology or diseases such as glaucoma remains undetermined. Increased knowledge of the proteins present in aqueous humor from POAG patients may provide clues to improve our understanding of the disease processes involved and how they interact with each other. Accordingly, the aims of this study were to compare the concentrations of selected proteins from these studies, including several not previously analysed in eye diseases, in aqueous humor samples obtained from a well-defined cohort of 19 POAG patients against 18 non-glaucomatous cataract samples. Subsequently, we sought to determine the extent of correlation between each of these proteins and relevant clinical descriptors including age, IOP, field of vision (quantified by Humphrey’s visual field pattern standard deviation (PSD) score and mean deviation (MD)), optic cup/disc ratio (CDR) and disease duration since commencing treatment. Here, we report the concentrations of six aqueous humor proteins, and identify significant correlations of age with Uteroglobin and FLRG specific to the POAG group, as well as a correlation of HGF with POAG disease duration.

## Methods

Patient eligibility and recruitment: This study was approved by the Health and Medical Human Research Ethics Committee Tasmania (H0013264), and executed in adherence to the tenets of the Declaration of Helsinki. All participants were recruited through Tze’Yo Toh at the Launceston Eye Institute and gave written consent with regards to donation and use of aqueous humor samples. POAG was diagnosed based on characteristic optic disc cupping, corresponding visual field loss, and retinal nerve fibre layer thinning, regardless of the presenting IOP. The anatomy of the drainage angle was assessed by gonioscopic examination. Non-glaucomatous cataract patients (referred to herein as the cataract group) were recruited to serve as a control for this study. POAG patients who had previously had a trabeculectomy or vitrectomy were excluded from this study. Furthermore, POAG and cataract subjects were excluded if they had other retinal (such as diabetic retinopathy or age-related macular degeneration) or neurological disease.

Clinical descriptors including age, IOP and CDR were recorded for both patient groups. IOP was measured in all patients using a calibrated Goldmann Applanation tonometer. For POAG patients, only the latest treated IOP measurement taken during the consultation prior to the surgery was used for this study. Vertical CDR was estimated by one observer (Tze’Yo Toh), using a 60D lens during indirect slit lamp fundoscopy and further confirmed with an optic disc profile scan using Ocular Coherence Tomography. MD and PSD were included as measures for vision loss, but were not available for all patients (MD was recorded for 13/19 POAG and 5/18 cataract patients, PSD for 19/19 POAG and 6/18 cataract patients). Furthermore, disease duration since commencing treatment was noted for POAG patients at the time AH samples were collected.

All POAG patients recruited were receiving IOP-lowering eye drops, in the form of monotherapy, or a combination of up to four of the following compounds: Timolol (beta-blocker), Bimatoprost, Tafluprost, Latanoprost, Travoprost (prostaglandin derivatives), Brimonidine (alpha 2 agonist) and Brinzolamide (carbonic anhydrase inhibitor).

Aqueous humor collection: Aqueous humor samples (50–100 μL) were collected from 19 patients with POAG during routine cataract surgery. Aqueous humor was also collected from 18 non-glaucomatous patients undergoing routine cataract surgery to serve as a control for this study [16, 23]. For all samples, aqueous humor was collected from the centre of the anterior chamber by paracentesis at the beginning of surgery, immediately frozen at − 20 °C, and transferred to − 80 °C within 48 h, where they were stored for analysis.

Multiplex immunoassay: We used discovery-based proteomic studies of AH in combination with other relevant scientific literature to select 30 proteins for inclusion in a custom magnetic bead-based multiplex immunoassay (R&D Systems, Inc., Minneapolis, MN) to enable simultaneous measurement of each protein in aqueous humor samples. The 30 proteins included were: Angiopoietin-1, Angiopoietin-2, BMP-2, BMP-4, BMP-9, CCL27/CTACK, CHI3L1/YKL-40, Collagen IV alpha 1, Cripto-1, DcR3, EGF, Endoglin/CD105, Endothelin-1, Epo, FLRG, Follistatin, Growth Hormone, HGF, IGFBP-1, IGFBP-3, IL-6, IL-9, LIF, MFG-E8, MIF, P-Selectin, Thrombospondin-2, Uteroglobin, VCAM-1 and vWF-A2. Some of these proteins (endothelin-1, HGF, EPO, MIF) have previously been shown to be present in aqueous humor using immunoassay techniques, but there are scant or no subsequent studies reporting correlation to clinical descriptors [12, 24–28]. Others (e.g. thromobospondin-2, follistatin) have been shown to be altered in animal [29] or cell culture-based [30] models of glaucoma, but there are no reports of their levels in aqueous humor. We also selected several proteins (e.g. CHI3L1, CTACK, Cripto-1, DcR3, Endoglin, uteroglobin, FLRG, MFG-E8, P-selectin) that have been identified as being present in aqueous humor, but for which there is a paucity of studies characterising their involvement in glaucoma.

The assay was performed in accordance with manufacturer's instructions on a Bio-Plex 200 System (Bio-Rad Laboratories, Inc., Hercules, CA). Aqueous humor dilutions with assay diluent were kept to a minimum, with dilution factors ranging from 1.5–5.5, sufficient to allow loading of 50 μl of diluted sample per assay well. Fluorescence intensity (FI) was measured and analysed using Bio-Plex Manager 6.0. The majority of concentrations out of range were below the detection limit for the relevant analyte, with the exception of MIF, which resulted in FIs above the highest standard for two cataract and three POAG samples. Readings out of range of the standard curve were excluded from all subsequent analyses. The concentration ranges of the standard curves and the number of samples in range for each analyte tested are given in Additional file 1: Table S1.

Normalisation to total protein concentration: Total aqueous humor protein concentration was measured using a BCA protein assay (Thermo Fisher Scientific, Waltham, MA). Aqueous humor samples were diluted 6-fold in ultrapure water and assessed as described in the manufacturer’s protocol. Individual analyte concentrations were normalised to total protein concentration for each sample prior to calculation of correlations as described below.

Statistical analyses: All statistical analyses were conducted with Prism 7 (GraphPad Software, San Diego, CA), using unpaired two-tailed tests with a significance threshold of *p* = 0.05. Differences in age, IOP, CDR and total protein concentration were assessed using unpaired T-tests, MD and PSD were evaluated with Mann-Whitney U tests due to non-Gaussian data distribution, and gender was tested using Fisher’s exact test. Analyte concentrations measured for POAG and cataract samples were grouped and the number of samples within range versus out of range of the standard curve were compared using Fisher’s exact test. Due to the non-normal distributions obtained for some analyte data sets, correlations with clinical descriptors were calculated using the non-parametric Spearman’s method (r_s_: Spearman’s correlation coefficient). To minimise identification of false associations in our data, Bonferroni’s method was used to correct for multiple testing of the analyte concentration data set across different analyses, resulting in an adjusted significance threshold of *p* = 0.0017 (conventional threshold of 0.05/30 protein analytes = adjusted threshold of *p* = 0.0017).

## Results

In this study, aqueous humor samples from 19 POAG and 18 non-glaucomatous cataract patients were analysed using a multiplex assay, to quantify the concentrations of 30 proteins reported to be present in aqueous humor [16, 17]. Clinical descriptors including age, IOP and CDR were collected for all patients and are presented in Table 1. MD and PSD were included as measures of vision loss; however, MD data was only available for 13 POAG and 5 cataract patients, and PSD for 6 cataract patients (Table 1). In addition, disease duration since commencing treatment was recorded for the POAG group (Table 1). There were no significant differences between cataract and POAG groups with regards to age (*p* = 0.335), gender (*p* = 0.313), IOP (*p* = 0.783) or total aqueous protein concentration (*p* = 0.077). Whilst the differences in MD and PSD were also non-significant (*p* = 0.846 and *p* = 0.0818, respectively), this is likely due to the lack of data for the majority of cataract patients. The difference in CDR was statistically significant, with a mean CDR of 0.78 in POAG compared to 0.40 in the cataract group (*p* < 0.0001). All patients in the POAG cohort were receiving IOP-lowering medication, with 63% (12/19) on a monotherapy regime of one prostaglandin derivative. The remaining patients received a combination of up to four compounds. All POAG patients were treated with a prostaglandin derivative, and 32% (6/19) were simultaneously prescribed with Timolol (β-blocker). A small percentage of patients received an α2-agonist (1/19) and/or a carbonic anhydrase inhibitor (2/19) in addition to the prostaglandin derivative and β-blocker. In this initial study, we have not attempted to assess differences in analyte concentrations due to specific medication regimes.

**Table 1:** Clinical data for non-glaucomatous cataract and POAG patients

Parameters	Cataract	POAG	*p*-value
Age (years; Mean ± SD)	66.5 ± 7.0	68.9 ± 7.9	0.335
Sample number (M/F)	18 (5/13)	19 (9/10)	0.313
IOP (Mean, ± SD)	17.9 ± 3.5	17.6 ± 3.4	0.783
MD (Median, IQR)	−3.1, −4.8 to − 0.9	− 3.6, − 4.8 to −2.0	0.846
PSD (Median, IQR)	1.63, 1.40–2.95	2.11, 1.69–5.26	0.082
CDR (Mean ± SD)	0.40, 0.22	0.78, 0.09	**< 0.0001**
Disease duration (years; Mean ± SD)	N/A	2.59, 2.15	N/A
AH total protein (mg/ml; Mean ± SD)	3.21 ± 0.88	3.77 ± 0.97	0.077

POAG: Primary open angle glaucoma; SD: standard deviation; M: male; F: female; IOP: intraocular pressure in mmHg; MD: mean deviation; IQR: interquartile range; PSD: Humphrey’s visual field pattern standard deviation; CDR: optic cup/disc ratio; N/A: not applicable; AH: aqueous humor. Statistical significance was assessed using Fischer’s exact test (gender), unpaired T-test (age, IOP, CDR, total protein) and Mann-Whitney U-test (MD, PSD) with *p* < 0.05 considered significant (highlighted in bold)

Out of the 30 proteins tested, 6 were detectable in ≥50% of samples in one or both groups: CHI3L1, FLRG, HGF, MIF, P-selectin and Uteroglobin (Table 2). The remaining 24 analytes were either not detected, or detected in only a small number of samples, and were therefore excluded from all subsequent analyses (see Additional file 1: Table S1). Of those proteins analysed further, CHI3L1 was present at the highest levels, with median concentrations above 65 ng/ml. FLRG and MIF were detected at intermediate levels, with median concentrations ranging from 3.6 to 6 ng/ml, whereas HGF, P-selectin and Uteroglobin were all quantified at median concentrations below 1 ng/ml.

**Table 2:** Aqueous humor analyte concentrations in non-glaucomatous cataract versus POAG

	Cataract			POAG			
Analyte	Median	IQR	In range	Median	IQR	In range	*p*-value
CHI3L1	65,171	53,191–91,124	18/18	83,122	65,958–95,018	19/19	1.000
FLRG	3614	2857–4412	16/18	4303	3679–5323	19/19	0.230
HGF	171.2	114–227	9/18	170.7	133–222	18/19	0.003
MIF	5592	4061–10,039	15/18	4809	3697–5830	15/19	1.000
P-selectin	791	691–874	9/18	927	803–1193	15/19	0.091
Uteroglobin	335	198–463	17/18	253	174–477	18/19	1.000

Median and interquartile range (IQR) calculated for data in range, reported as pg/ml. Significance was tested using the Fisher’s exact test for comparison of number of detected vs. undetected samples in each group. Following correction for multiple testing using Bonferroni’s method, a p-value of < 0.0017 was considered significant

The number of samples in range versus below the range of the standard curve were compared for each analyte using Fisher’s exact test. Whilst analysis revealed a difference for HGF (*p* = 0.003) between cataract (9/18 in range) and POAG (18/19 in range), the result did not pass correction for multiple testing (adjusted *p*-value threshold = 0.0017). In addition, no sample was consistently below the 5th or above the 95th percentile for all analytes reported.

### Significant correlation of FLRG and uteroglobin with age in POAG but not cataract

**Fig. 1:**
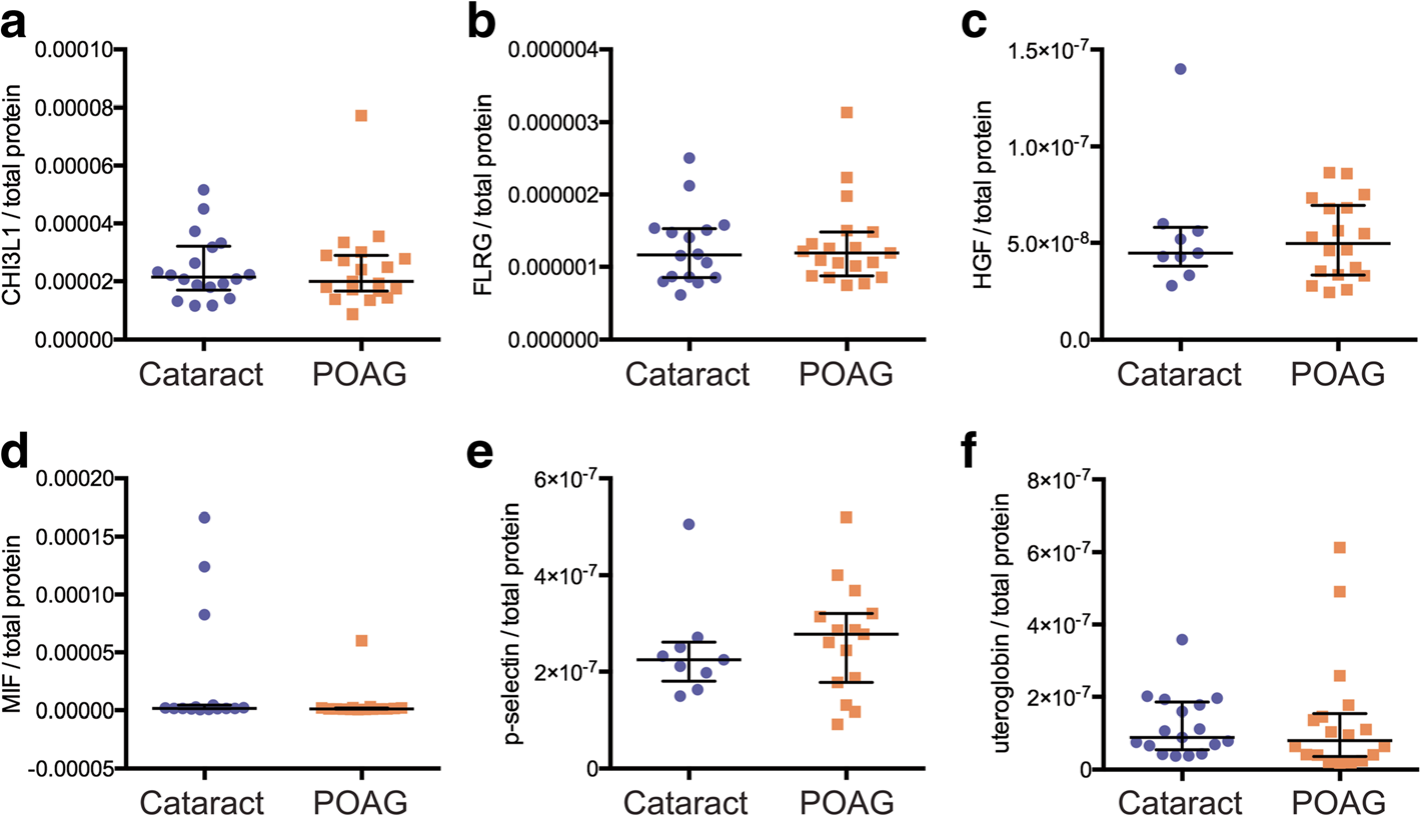
Normalised analyte distributions in cataract and primary open angle glaucoma (POAG) samples. Distribution of CHI3L1 (**a**), FLRG (**b**), HGF (**c**), MIF (**d**), P-selectin (**e**) and Uteroglobin (**f**) concentrations in aqueous humor normalised to total aqueous humor protein concentration from non-glaucomatous cataract (blue) and POAG (orange). Median and interquartile range are indicated

Prior to calculating correlations, analyte concentrations were normalised using total aqueous humor protein concentration. The total protein concentrations determined for POAG and cataract samples (mean concentrations of 3.77 and 3.21 mg/ml, respectively) did not differ significantly between groups (*p* = 0.077). Similarly, normalised analyte concentrations did not differ significantly between the POAG and cataract group (Fig. 1).

Correlations between normalised analyte concentrations and age were assessed for both patient groups (Table 3). Significant positive correlations were obtained with FLRG (r_s_ = 0.706, *p* = 0.0007) and Uteroglobin (r_s_ = 0.805, *p* < 0.0001) for POAG but not the cataract group (r_s_ = 0.475, *p* = 0.065 and r_s_ = 0.555, *p* = 0.022, respectively). Whilst further correlations were determined for CHI3L1 (r_s_ = 0.566, *p* = 0.012) and HGF (r_s_ = 0.642, *p* = 0.004) in POAG, these did not pass correction for multiple testing. No significant correlations were obtained between age and other analytes (all *p* ≥ 0.05).

**Table 3:** Correlation of measured analytes to age for non-glaucomatous cataract and POAG samples

	Cataract			POAG		
Analyte	r_s_	*p*-value	N	r_s_	*p*-value	N
CHI3L1	−0.066	0.794	18	0.566	0.012	19
FLRG	0.475	0.065	16	0.706	**0.0007**	19
HGF	0.170	0.662	9	0.642	0.004	18
MIF	0.404	0.135	15	0.159	0.570	15
P-selectin	−0.756	0.835	9	0.500	0.060	15
Uteroglobin	0.555	0.022	17	0.805	**< 0.0001**	18

Correlations of normalised analyte concentrations to age were determined using Spearman’s rank correlation. Following correction for multiple testing using Bonferroni’s method, a *p*-value of < 0.0017 was considered significant (highlighted in bold). r_s_: Spearman correlation coefficient. N: number of correlation pairs

### HGF correlated significantly with POAG disease duration since commencing treatment

Analyte concentrations were also assessed for correlations with CDR, IOP, PSD and MD for both patient groups, and with disease duration since commencing treatment in POAG only (Table 4 and Additional file 1: Tables S2-S5). A significant correlation was determined between HGF and disease duration (r_s_ = − 0.723, *p* = 0.0007, Table 4). Further correlations with disease duration were determined for CHI3L1 and FLRG (r_s_ = − 0.555, *p* = 0.014 and r_s_ = − 0.673, *p* < 0.002, respectively, Table 4), and CHI3L1 correlated with CDR in cataract (r_s_ = − 0.539, *p* = 0.021, Additional file 1: Table S3), however, they did not pass correction for multiple testing (adjusted *p*-value threshold = 0.0017). No correlations were obtained between IOP (Additional file 1: Table S2), MD (Additional file 1: Table S4) or PSD (Additional file 1: Table S5) and any analyte for either patient group.

**Table 4:** Correlation of measured analytes to disease duration for POAG samples

	Disease duration*		
Analyte	r_s_	*p*-value	N
CHI3L1	−0.555	0.014	19
FLRG	−0.673	0.002	19
HGF	−0.723	**0.0007**	18
MIF	−0.306	0.246	15
P-selectin	−0.312	0.226	15
Uteroglobin	−0.377	0.123	18

Correlations of normalised analyte concentrations to disease duration (*in years since commencing treatment) were determined using Spearman’s rank correlation. Following correction for multiple testing using Bonferroni’s method, a *p*-value of < 0.0017 was considered significant (highlighted in bold). r_s_: Spearman correlation coefficient. N: number of correlation pairs

## Discussion

In this present study, aqueous humor samples were collected and analysed from 19 POAG and 18 non-glaucomatous cataract patients as controls. Out of the 30 analytes measured, 6 were quantified in sufficient samples to allow for further analysis (CHI3L1, FLRG, HGF, MIF, P-selectin and Uteroglobin), some of which have not previously been assessed with regards to eye physiology or disease. The concentrations obtained for HGF and MIF are consistent with existing literature [12, 26, 31] and to the best of our knowledge, no quantitative measures of CHI3L1, FLRG, P-selectin or Uteroglobin have been reported in aqueous humor. Four of these proteins are directly linked to inflammation: P-selectin and MIF are both pro-inflammatory mediators, with P-selectin mediating leukocyte-endothelium adhesion [32], and MIF suppressing the anti-inflammatory and immunosuppressive effects of glucocorticoids [33]. CHI3L1 exerts its pro-inflammatory effects at least in part by inhibiting apoptosis of T-cells, macrophages and eosinophils [34]. In contrast, uteroglobin has anti-inflammatory effects [35, 36]. Furthermore, HGF is involved in tissue repair [26] and FLRG acts as an inhibitor to members of the TGFβ superfamily [37]. Whilst no significant differences were found between normalised analyte concentrations, significant correlations of specific analytes with disease descriptors were obtained, which are discussed below.

A positive correlation was determined for Uteroglobin with age in POAG but not cataract samples, which may indicate an increased need for anti-inflammatory activity in the ageing glaucomatous TM. Uteroglobin is primarily known for its association with various allergic and inflammatory lung diseases [38], where it exerts an anti-inflammatory effect by supressing various inflammatory mediators, including INFγ, PLA2 and TNFα [35, 36]. In addition, Uteroglobin plays a protective role against oxidative stress [39]. In eosinophilic chronic rhinosinusitis, uteroglobin suppresses the expression of pro-inflammatory CHI3L1 [40]. CHI3L1 is a commonly used TM cell marker [41–43], although it is only expressed by TM cells in the most anterior and posterior regions of the TM tissue [42], which may reflect areas subject to the greatest levels of tissue remodelling within the TM. Interestingly, in this study, uteroglobin and CHI3L1 correlated in the POAG group but not in the cataract group (POAG *p* = 0.006, r_s_ = 0.624; cataract *p* = 0.126, r_s_ = 0.387), which may suggest that a similar mechanism could be occurring in POAG, however, this correlation did not pass correction for multiple testing.

HGF levels were negatively associated with disease duration since commencing treatment in POAG samples, indicating a reduction of HGF over time, which may be linked to or independent from treatment for hypertension. HGF plays a role in tissue repair, and is therefore closely linked to inflammation [26]. HGF stimulates proliferation, migration and differentiation of many cell types, including TM cells [44], and can stimulate MMP activity in endothelial cells [45]. In this study, the number of samples where HGF was above a set threshold of detection was analysed between the POAG and cataract group (Table 2, *p* = 0.003); whilst this comparison did not pass correction for multiple testing, the result is in line with published literature, showing a significant increase in HGF in glaucomatous aqueous humor in relation to cataract samples [26]. It has been suggested that elevated HGF levels in glaucomatous aqueous humor may play a compensatory role, by increasing aqueous humor outflow, or aiding in repairing TM damage [26]. The correlation suggests that this compensation may be lost over time.

Similar to uteroglobin, FLRG correlated positively with age in POAG but not cataract. FLRG is a secreted glycoprotein highly homologous to follistatin [46] that binds to and thereby inactivates members of the TGFβ superfamily, including activin A and BMP2, by disabling their ability to interact with cell surface receptors [37]. Interestingly, whilst FLRG was measurable, follistatin was not detected in any of the samples analysed in this study. Within the anterior segment, FLRG may be involved in the regulation of BMP2-induced calcification of the trabecular ECM, which has been suggested to occur with age and to be more prominent in glaucomatous TM [8]. The correlation of FLRG with age may indicate a greater need for BMP-2 inhibition, due to increased calcification. BMP2-induced calcification of the TM has been shown to directly lead to elevated IOP in a POAG rat model [8] and also agrees with existing reports of increased TM stiffness in POAG [7].

Whilst several correlations were determined between analytes and disease descriptors at a significance threshold of *p* = 0.05, six out of nine did not pass correction for multiple testing (adjusted *p* = 0.0017), but may do so in other appropriately powered studies. Although this study did not include a replication cohort, each analyte measured was selected from either discovery-based proteomic studies or immunoassay-based results reported by other groups [12, 16, 24–28]. Despite this, we were unable to detect many of the proteins included in this study at levels above the lower limit of our standard curves. Aside from technical limitations of our chosen multiplex immunoassay, contributing factors many include the variation in proteins identified across multiple proteomic studies [16, 20], as well as the wide spread of specific analyte concentrations observed between aqueous humor samples from different individuals, as reported in some studies [47].

It is important to note that some topical treatments commonly used to treat ocular hypertension, such as latanoprost and brimonidine, may contribute to aqueous inflammation [48, 49]. Whilst the potential effects of such medication on the protein concentrations discussed here are not specifically known, altered aqueous humor concentrations of other proteins have been reported [50]. Although there were no differences between the normalised analyte concentrations measured in POAG and cataract for the 6 analytes studied here, the potential contributions from patient medications to the associations reported here cannot be excluded.

## Conclusion

In conclusion, this study has expanded our knowledge of aqueous humor composition by providing quantitative measures for four proteins previously undetermined for aqueous humor. The correlations of Uteroglobin and FLRG with age in POAG may suggest an increased need for compensation of inflammatory and calcifying activity in the ageing glaucomatous TM to maintain functionality, but at present it is unclear whether these proteins play a causative or compensatory role. If any or all of these proteins are to have clinical utility, be it as a diagnostic biomarker or therapeutic target, further research is needed to define their contributions to TM cell physiology, particularly in respect to aqueous humor inflammation and TM outflow resistance, and to determine if these proteins have roles in the onset and progression of POAG.

## Additional file

Additional file 1: This file contains Supplementary Tables S1 to S5, which report correlations to relevant clinical descriptors in the cataract and POAG groups. (DOCX 32 kb)

## Abbreviations

CDR: Cup-to-disk ratio
ECM: Extracellular matrix
FI: Fluorescence intensity
IOP: Intraocular pressure
MD: Mean deviation
POAG: Primary open angle glaucoma
PSD: Humphrey’s visual field pattern standard deviation
TM: Trabecular meshwork
