# Supplementary material for: Uteroglobin and FLRG concentrations in aqueous humor are associated with age in primary open angle glaucoma patients
Source: BMC Ophthalmol. 2018 Feb 27;18:57. doi: 10.1186/s12886-018-0723-4 (PMC5828060; doi:10.1186/s12886-018-0723-4)
Supplement: Supplementary file 1 — This file contains Supplementary Tables S1 to S5, which report correlations to relevant clinical descriptors in the cataract and POAG groups. (DOCX 32 kb) [file 12886_2018_723_MOESM1_ESM.docx]

Title:

Uteroglobin and FLRG concentrations in aqueous humor are associated with age in primary open angle glaucoma patients

Authors:

Esther L Ashworth Briggs (BSc Hons)^1^, Tze’Yo Toh (FRANZCO)^2^, Rajaraman Eri (PhD)^1^, Alex W. Hewitt (FRANZCO, PhD)^1,3^, Anthony L. Cook (PhD) ^1,4^

^1^School of Health Sciences, University of Tasmania

^2^Launceston Eye Institute and Launceston Eye Doctors, Tasmania

^3^Centre for Eye Research Australia, University of Melbourne, Victoria.

^4^Wicking Dementia Research and Education Centre, University of Tasmania

Email addresses:

[esther.ashworthbriggs@utas.edu.au](mailto:esther.ashworthbriggs@utas.edu.au)

[idoctoh@gmail.com](mailto:idoctoh@gmail.com)

[rajaraman.eri@utas.edu.au](mailto:rajaraman.eri@utas.edu.au)

[alex.hewitt@utas.edu.au](mailto:alex.hewitt@utas.edu.au)

[anthony.cook@utas.edu.au](mailto:anthony.cook@utas.edu.au)

Correspondence to:

Anthony L Cook, Ph.D., Wicking Dementia Research and Education Centre, University of Tasmania, Private Bag 143, Hobart, Australia 7001; Phone: +61 3 6226 6964; FAX: +61 3 6226 4880; email: [anthony.cook@utas.edu.au](mailto:anthony.cook@utas.edu.au)

SUPPLEMENTARY TABLES

| **Additional file 1: Table S1. Standard curve ranges of multiplex assays** | | | | |
| --- | --- | --- | --- | --- |
| **Analyte** | **HGNC code** | **Range** | **Cataract detected/total** | **POAG detected/total** |
| Angiopoietin-1 | ANGPT1 | 127.5 - 30980 | 0/18 | 0/19 |
| Angiopoietin-2 | ANGPT2 | 113.0 - 27450 | 0/18 | 0/19 |
| BMP-2 | BMP2 | 32.4 - 7860 | 0/18 | 0/19 |
| BMP-4 | BMP4 | 48.3 - 11730 | 0/18 | 0/19 |
| BMP-9 | GDF2 | 5.0 - 1200 | 0/18 | 0/19 |
| CCL27/CTACK | CCL27 | 7.5 - 1830 | 0/18 | 0/19 |
| **CHI3L1/YKL-40** | **CHI3L1** | **368.2 - 89460** | **18/18** | **19/19** |
| Collagen IV alpha 1 | COL4A1 | 122.9 - 29860 | 1/18 | 1/19 |
| Cripto-1 | CFC1 | 40.7 - 9900 | 0/18 | 0/19 |
| DcR3 | TNFRSF6B | 1031.2 - 250580 | 0/18 | 0/19 |
| EGF | EGF | 17.8 - 4320 | 0/18 | 0/19 |
| Endoglin/CD105 | ENG | 463.4 - 112610 | 0/18 | 0/19 |
| Endothelin-1 | EDN1 | 31.9 - 7760 | 0/18 | 0/19 |
| Epo | EPO | 456.8 - 111000 | 0/18 | 0/19 |
| **FLRG** | **FSTL3** | **961.6 - 233660** | **16/18** | **19/19** |
| Follistatin | FST | 2674.9 - 650000 | 0/18 | 0/19 |
| Growth Hormone | GH1 | 76.1 - 18500 | 0/18 | 0/19 |
| **HGF** | **HGF** | **43.2 - 10500** | **9/18** | **18/19** |
| IGFBP-1 | IGFBP1 | 163.3 - 39690 | 6/18 | 8/19 |
| IGFBP-3 | IGFBP3 | 2824.7 - 686400 | 0/18 | 0/19 |
| IL-6 | IL6 | 13.9 - 3370 | 0/18 | 0/19 |
| IL-9 | IL9 | 3012.4 - 732000 | 0/18 | 0/19 |
| LIF | LIF | 68.1 - 16550 | 0/18 | 0/19 |
| MFG-E8 | MFGE8 | 391.8 - 95200 | 2/18 | 1/19 |
| **MIF** | **MIF** | **781.9 - 190000** | **15/18** | **15/19** |
| **P-Selectin** | **SELP** | **223.3 - 54270** | **9/18** | **15/19** |
| Thrombospondin-2 | THBS2 | 349.2 - 84860 | 0/18 | 0/19 |
| **Uteroglobin** | **SCGB1A1** | **30.5 - 7400** | **17/18** | **18/19** |
| VCAM-1 | VCAM1 | 7440.7 - 1808100 | 6/18 | 8/19 |
| vWF-A2 | VWF | 97.1 - 23600 | 0/18 | 0/19 |
| HGNC: HUGO Gene Nomenclature Committee. Range: Concentration range of standard curve in pg/ml. POAG: Primary open angle glaucoma. Analytes detected in more than 50% of samples in at least one patient group are highlighted in bold and were further analysed (see Tables 2-4). | | | | |

| **Additional file 1: Table S2. Correlation of measured analytes to IOP for non-glaucomatous cataract and POAG samples** | | | | | | | |
| --- | --- | --- | --- | --- | --- | --- | --- |
|  | **Cataract** | |  |  | **POAG** | |  |
| **Analyte/ratio** | **r_s_** | **p-value** | **N** |  | **r_s_** | **p-value** | **N** |
| CHI3L1 | 0.050 | 0.845 | 18 |  | -0.416 | 0.076 | 19 |
| FLRG | -0.040 | 0.826 | 16 |  | -0.044 | 0.857 | 19 |
| HGF | -0.417 | 0.236 | 9 |  | -0.402 | 0.098 | 18 |
| MIF | -0.167 | 0.480 | 15 |  | -0.326 | 0.183 | 15 |
| P-selectin | -0.369 | 0.275 | 9 |  | -0.398 | 0.106 | 15 |
| Uteroglobin | -0.379 | 0.113 | 17 |  | -0.055 | 0.827 | 18 |
| Correlations of normalised analyte concentrations to intraocular pressure (IOP) were determined using Spearman’s rank correlation. Following correction for multiple testing using Bonferroni’s method, a p-value of <0.0017 was considered significant. r_s_: Spearman correlation coefficient; N: number of correlation pairs. | | | | | | | |

| **Additional file 1: Table S3. Correlation of measured analytes to CDR for non-glaucomatous cataract and POAG samples** | | | | | | | |
| --- | --- | --- | --- | --- | --- | --- | --- |
|  | **Cataract** | | |  | **POAG** | | |
| **Analyte** | **r_s_** | **p-value** | **N** |  | **r_s_** | **p-value** | **N** |
| CHI3L1 | -0.539 | 0.021 | 18 |  | -0.004 | 0.988 | 19 |
| FLRG | -0.249 | 0.224 | 16 |  | -0.091 | 0.711 | 19 |
| HGF | -0.292 | 0.310 | 9 |  | -0.110 | 0.665 | 18 |
| MIF | -0.213 | 0.322 | 15 |  | -0.139 | 0.560 | 15 |
| P-selectin | -0.588 | 0.635 | 9 |  | -0.091 | 0.648 | 15 |
| Uteroglobin | -0.191 | 0.325 | 17 |  | -0.163 | 0.520 | 18 |
| Correlations of normalised analyte concentrations to cup-to-disc ratio (CDR) were determined using Spearman’s rank correlation. Following correction for multiple testing using Bonferroni’s method, a p-value of <0.0017 was considered significant. r_s_: Spearman correlation coefficient; N: number of correlation pairs. | | | | | | | |

| **Additional file 1: Table S4. Correlation of measured analytes to MD for non-glaucomatous cataract and POAG samples** | | | | | | | |
| --- | --- | --- | --- | --- | --- | --- | --- |
|  | **Cataract** | |  |  | **POAG** | |  |
| **Analyte/ratio** | **r_s_** | **p-value** | **N** |  | **r_s_** | **p-value** | **N** |
| CHI3L1 | 0.100 | 0.950 | 5 |  | 0.187 | 0.541 | 13 |
| FLRG | 0.400 | 0.750 | 4 |  | -0.445 | 0.130 | 13 |
| HGF | 0.500 | >0.999 | 3 |  | -0.252 | 0.430 | 12 |
| MIF | 0.600 | 0.417 | 4 |  | -0.050 | 0.912 | 9 |
| P-selectin | N/A | N/A | 1 |  | -0.119 | 0.716 | 12 |
| Uteroglobin | -0.200 | 0.783 | 5 |  | -0.336 | 0.287 | 12 |
| Correlations of normalised analyte concentrations to mean deviation (MD) were determined using Spearman’s rank correlation. Following correction for multiple testing using Bonferroni’s method, a p-value of <0.0017 was considered significant. r_s_: Spearman correlation coefficient; N: number of correlation pairs; N/A: not available. | | | | | | | |

| **Additional file 1: Table S5. Correlation of measured analytes to PSD for non-glaucomatous cataract and POAG samples** | | | | | | | |
| --- | --- | --- | --- | --- | --- | --- | --- |
|  | **Cataract** | |  |  | **POAG** | |  |
| **Analyte/ratio** | **r_s_** | **p-value** | **N** |  | **r_s_** | **p-value** | **N** |
| CHI3L1 | 0.086 | 0.919 | 6 |  | 0.260 | 0.283 | 19 |
| FLRG | 0.700 | 0.233 | 5 |  | 0.493 | 0.032 | 19 |
| HGF | 1.000 | 0.333 | 3 |  | 0.536 | 0.022 | 18 |
| MIF | -0.600 | 0.350 | 5 |  | 0.136 | 0.630 | 15 |
| P-selectin | N/A | N/A | 2 |  | 0.186 | 0.504 | 15 |
| Uteroglobin | -0.257 | 0.658 | 6 |  | 0.212 | 0.399 | 18 |
| Correlations of normalised analyte concentrations to Humphrey’s visual field pattern standard deviation (PSD) were determined using Spearman’s rank correlation. Following correction for multiple testing using Bonferroni’s method, a p-value of <0.0017 was considered significant. r_s_: Spearman correlation coefficient; N: number of correlation pairs; N/A: not available. | | | | | | | |
